# Vibrotactile stimulus duration threshold for perception of pulse to vibration transition

**DOI:** 10.1038/s41598-025-85778-6

**Published:** 2025-02-11

**Authors:** Byron Remache-Vinueza, Andrés Trujillo-León, Fernando Vidal-Verdú

**Affiliations:** 1 Departamento de Electrónica, Universidad de Málaga, 29071 Málaga, Spain; 2 Instituto Universitario de Investigación en Ingeniería Mecatrónica y Sistemas Ciberfísicos, IMECH.UMA, 29590 Campanillas, Spain; 3 Facultad de Ingenierías, Ingeniería Industrial, Universidad Tecnológica Indoamérica, 170103 Quito, Ecuador

**Keywords:** Human behaviour, Electrical and electronic engineering

## Abstract

This study investigates the minimum stimulus duration required to perceive the transition from pulse to vibration sensations, a critical parameter for optimizing information transmission via haptic interfaces such as smartphones, tablets, smartwatches, game consoles, and sensory substitution systems. Efficient transmission relies on minimizing stimulus duration, enabling more information to be conveyed in less time. A preliminary experiment established intensity perception thresholds—the minimum vibration intensities detectable—at 40, 80, 150, 250, 300, and 590 Hz, frequencies primarily activating the Pacinian (Rapid Adapting II) psychophysical channel. Subsequently, 35 participants determined the minimum durations needed to perceive the transition from pulse to vibration sensations across this frequency range. Results revealed a consistent minimum duration of approximately 30 ms, contrasting with findings in audition, where shorter durations suffice at higher frequencies, but aligning with prior studies in tactile perception.

## Introduction

Human beings perceive vibrations through the auditory system as audible sound and through the sense of touch via mechanoreceptors located in the skin, muscles, bones, and ligaments^1^. Investigating the specifics of vibration perception is essential, as these stimuli are often synthesized for use in haptic interfaces, particularly in multimodal applications for human-computer interaction (HCI)^2–4^. Common examples include video game consoles, smartphones, smartwatches, virtual and augmented reality environments, haptic music players^5,6^, and applications in medical and therapeutic procedures^7–9^.

Tactile perception of vibration sensations depends on the type of mechanoreceptors activated during stimulation. The Slowly Adapting I (SA-I) channel detects vibrations at frequencies lower than or equal to 6.3 Hz. Low-frequency vibrations between 16 and 32 Hz activate the Rapidly Adapting I (RA-I) channel, producing a fluttering sensation - perceived as rhythmic beats at a frequency matching the vibration but distinct from typical vibration sensations^10^. The Rapidly Adapting II (RA-II), or Pacinian, channel is responsible for high-frequency vibration perception in the range of 35–500 Hz^1^.

The perception of vibrotactile stimuli, defined as the sensation caused by vibrations, has been studied for decades^11^. Research shows that intensity perception thresholds depend on vibration frequency, reaching a minimum at approximately 250 Hz, with sensitivity decreasing at frequencies below and above this point^12,13^. The vibrotactile perception threshold contour, which connects perception thresholds across frequencies, shifts depending on variables such as age, gender, and body location^14^. Understanding these effects enables the design of vibrotactile stimuli tailored to individual characteristics.

Temporal aspects of vibrotactile stimulation also significantly influence perception. For instance, efficient information transmission through vibrotactile spatio - temporal patterns, such as pulses and vibrations, depends on factors like minimum stimulus duration required for perception^15–17^. Additionally, temporal masking occurs when two or more stimuli are presented in close temporal proximity, where one stimulus may obscure the other. This effect depends on factors such as the intensity and frequency of the masker stimulus and the inter-stimulus onset asynchrony (ISOA)^18,19^. Notably, temporal masking does not occur across psychophysical channels; stimuli in one channel do not affect perception in another^20,21^.

Adaptation is another temporal effect in vibrotactile perception. Prolonged stimulation, such as a 10-minute duration, can reduce sensitivity, increasing the intensity perception threshold of subsequent stimuli^14^. This adaptation effect is also channel-specific and does not transfer across psychophysical channels^20^. Conversely, the summation effect, characterized by a decrease in perception thresholds as stimulus duration increases (below 1000 ms), is present only in the RA-II channel^14,22^. Temporal resolution studies have shown that individuals can discriminate pairs of stimuli separated by as little as 8–12 ms, although this resolution decreases with lower stimulus intensity^23^.

However, an unsolved question arises regarding the minimum duration required for the perception of vibration sensations. Even though it is well established that two channels mediate the perception of fluttering and vibration sensations, with specific frequency ranges evoking these sensations, it remains unclear whether the vibration attributes of a stimulus disappear as its duration approaches zero. This hypothesis stems from studies in the auditory modality, where researchers have identified two sensation transition thresholds as duration varies: the *click - pitch* and *pitch - tone* transitions^24^. Roads C., in his book *Microsound*, discusses how audible vibrations, or sound, occur on different time scales, and how human perception of sound changes depending on the scale. He also notes that the perception of pitch in auditory stimuli depends on frequency. These hypotheses are further supported by Doughty J. and Garner W.^24^, who reported that for a peak frequency of 4000 Hz, the *click*-*pitch* transition occurs with a stimulus duration of approximately 4.1 ms.

In a related investigation, Burton R. asked experienced musicians to compare sine wave tones with piano tones^25^. He found that durations around 8 ms were necessary for pitch differentiation. Burton also observed that low frequencies require longer durations for pitch perception, and that the ability to perceive pitch is influenced by the frequency content of the comparison stimulus, such as the timbre of piano tones.

In this context, one may infer that individuals should be able to recognize the *pulse - vibration* sensation transition with the sense of touch, analogous to the *click - pitch* sensation transition in audition. But it is important to consider the characteristics of the sense of touch previously described. Therefore, we divided our investigation into two stages:

1. In a pilot experiment (See “Pilot experiment” Section), we normalized the perception thresholds with respect to the frequency using the *one down–one up* method, obtaining the intensity perception threshold contour that corresponds to our experimental setup (See “Main experimental procedure” Section). This first step is important to ensure good perception of vibrotactile stimulation along the selected vibration frequency range: 40 Hz, 80 Hz, 150 Hz, 250 Hz, 300 Hz, and 590 Hz. In addition, considering the complex multichannel nature of the sense of touch, vibrotactile stimulation was focused on frequencies above 35 Hz to avoid fluttering sensations, i.e. activation of the RA-I channel, and ensure vibration sensations only. In this stage, we also determined a suitable duration for the perception of vibration sensations according to feedback from the participants.
2. In the main experiment (See Section Main experimental procedure” Section), we calibrated the intensity perception threshold contour according to feedback from each participant, to not only warrant optimal perception of vibrotactile stimulation, but also to minimize effects like those related to age and gender^13^. Then, the participant took part in a training session to familiarize themselves with the user interface and understand the experimental procedure. Finally, each participant evaluated the *pulse-vibration* sensation transition using the method of *adjustment* for the vibration frequency range described above.

The remainder of this article is organized as follows: “Related work” Section presents the foundation and main goals of the investigation. “Methods” Section outlines the materials and methods used to validate the proposed hypothesis. In “Results” Section, the experimental results are presented, followed by a comprehensive analysis of these findings in “Discussion” Section, where they are contrasted with those found in the literature. Finally, “Conclusions” Section provides a summary of the main findings, a response to the research question, and suggestions for future work.

## Related work

The minimum duration of vibrotactile stimuli for frequency discrimination was investigated by Cohen and Kirman^26^. The authors used discrete values for stimulus duration: 30, 50, 100, and 200 ms. Participants compared a standard stimulus with a comparison stimulus. The standard stimulus had a frequency of 100 Hz, while the comparison stimuli had frequencies of 70, 80, 90, 100, 120, and 130 Hz. The researchers found that the ability to discriminate frequencies remained approximately constant for durations between 50 and 200 ms. Cohen and Kirman highlight that a significant decrease in frequency discrimination ability was observed for durations between 50 and 30 ms, but they attribute this effect to a practice effect. The authors note that these results contradict those found in the auditory modality, where frequency discrimination consistently decreases as duration decreases in the same range.

Similarly, Kobayashi D., in his work *Study on Perception of Vibration Rhythms*^27^, designed an experimental procedure to investigate the minimum duration for vibration perception in rhythmic contexts, i.e., consecutive presentation of vibrotactile stimuli separated by a temporal gap. The gaps were established at 200, 400, 500, 700, 800, and 1000 ms, while the stimulus duration was initially set at 300 ms and then reduced in steps of 5, 10, or 50 ms. Kobayashi concludes that 50 ms stimulus durations are necessary for participants to perceive the vibrations in vibrotactile rhythmic patterns. However, the author does not specify the testing frequencies.

Moreover, Cui and Mousas^28^ designed an experiment to determine the just noticeable difference (JND) for three vibration properties: duration, frequency, and intensity, using a commercial vibrotactile interface. For duration, the researchers used a reference stimulus with a duration of 500 ms, a frequency of 100 Hz, and an intensity of 1 m/s. The durations of the comparison stimuli were 100, 200, 300, 400, 600, 700, 800, 900, and 1000 ms. According to the results, the JND was between 200 and 300 ms for a positive comparison (i.e., the stimulus with shorter duration presented first), while the JND was slightly longer than 100 ms for a negative comparison (i.e., the stimulus with longer duration presented first). The authors suggest that participants are more sensitive to negative variations than to positive variations.

Finally, Bochereau et al. reported the influence of vibrotactile stimulus duration on perceived intensity in the context of pink noise vibrations^29^. The duration of the stimuli ranged from 100 ms to 700 ms, and the authors found that the perceived amplitude of vibrotactile stimuli decreased as duration increased.

### Goal of this investigation

As presented in previous sections, investigating the perception of vibratory sensations through touch is of significant relevance to the scientific community. However, following a systematic review of the literature, the authors of this study found no reports addressing the influence of stimulus duration on perceived vibration sensations. In this context, we propose an experimental design inspired by the work of Doughty and Garner^24^, aimed at determining the minimum duration of vibrotactile stimuli at which individuals perceive the *pulse - vibration* sensation transition. To achieve this, we will employ the method of adjustment.

## Methods

### Participants

In the pilot experiment, eight healthy adult volunteers were recruited from the University of Malaga (Spain), consisting of 3 females and 5 males, aged between 22 and 58 years old (*M* = 41 years, *SD* = 11 years).

For the main experiment, participants were recruited from the Indoamerican University (Quito, Ecuador). In total, 35 healthy adult volunteers participated, with 16 females and 19 males. The age range of participants was from 19 to 60 years old (*M *= 30 years, *SD* = 9 years).

All participants were right-handed, and none reported any tactile or auditory impairments. Informed consent was obtained from all participants.

### Vibrotactile interface

**Fig. 1:**
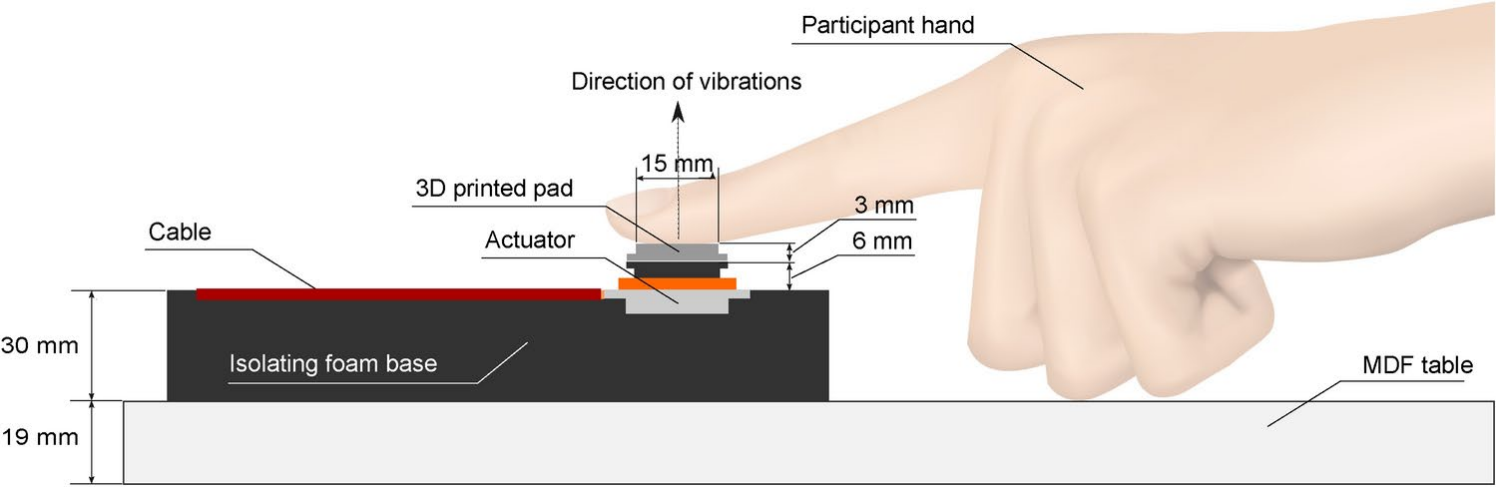
Vibrotactile interface (not to scale).

Monophonic audio signals, synthesized in MATLAB, were routed from the left channel of the audio output of an Asus ZenBook UX425E to the left audio input of a Fosi TDA7498E audio stereo amplifier. The left audio output of the amplifier was then sent to the vibrotactile interface. The vibrotactile interface featured an 8 ohm, 2 Watt TEAX19001-8 voice coil tactor from Tectonic, along with isolating foam and a 3D-printed PLA pad with a 15 mm diameter (1.8 cm° circular contact area), which enabled effective perception of vibrations^13^. The vibrotactile interface is depicted in Fig. 1.

### Stimuli

The signal in all trials was a sine wave, with a frequency sampling rate set to 10 kHz. The frequencies selected for this investigation were 40 Hz, 80 Hz, 150 Hz, 250 Hz, and 590 Hz, which represent a frequency range commonly used in studies by Verillo et al.^12^. Tactile stimulation in this range primarily activates the Pacinian channel, evoking vibration sensations^1^.

### Experimental setup

**Fig. 2:**
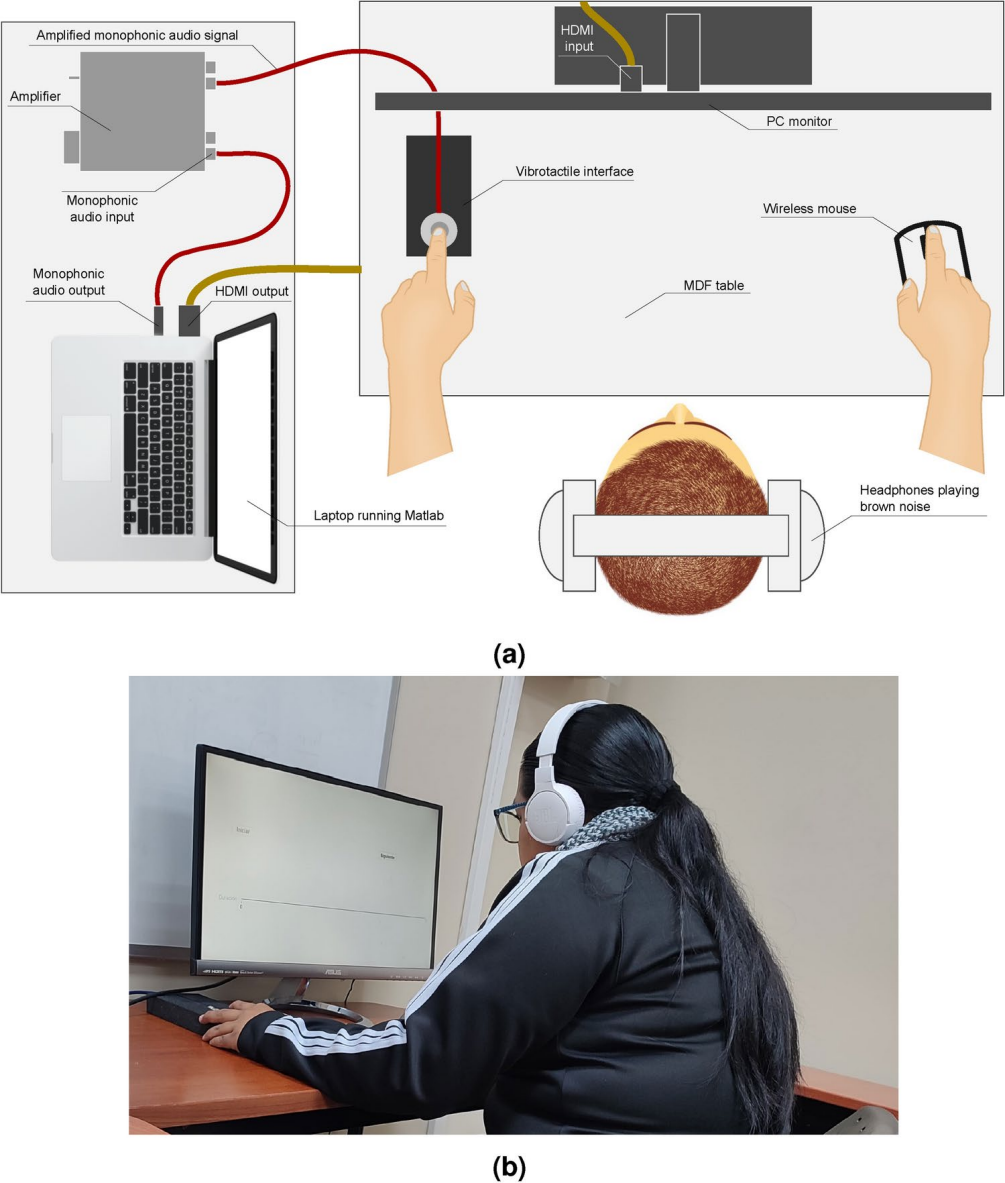
(**a**) Setup for experimentation. (**b**) A participant in the main experiment. Informed consent was obtained from the participant to capture her photograph and include it in this manuscript..

The experimental setup included a chair without armrests, a desk, and a visual user interface designed in MATLAB App Designer, displayed on an Asus MX239H PC monitor. The user controlled the interface using a wireless KlipXtreme KMW-390 mouse. Brown noise was played via Bluetooth from a mobile phone through JBL TUNE660NC headphones. The setup is shown in Fig. 2a.

### Experimental procedures

All experimental procedures were reviewed and approved by the *Ethics Committee of the University of Malaga*, Spain (Register No. CEUMA 58-2022-H, July 12, 2022). Additionally, the *Ethics Committee of the Indoamerican University*, Ecuador, validated the protocol. All procedures adhered to the principles outlined in the *Declaration of Helsinki*. At no point did the vibrotactile stimulation reach or exceed the pain threshold.

#### Pilot experiment

**Fig. 3:**
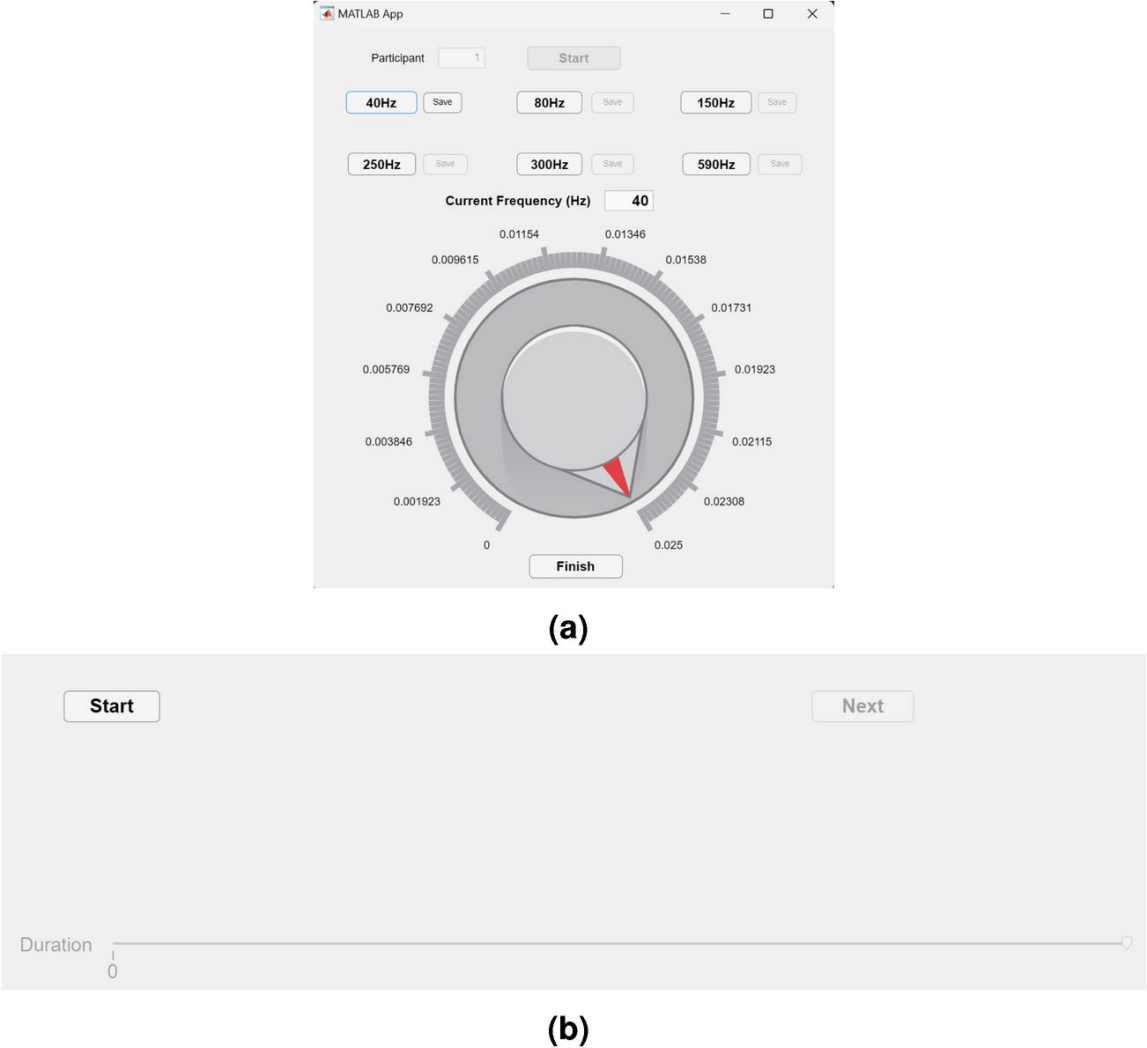
User interface for (**a**) the experimenter in the pilot study and (**b**) for the participant in the main experiment.

A pilot experiment was conducted to determine the intensity perception threshold contour and the appropriate maximum stimulus duration. The intensity perception threshold contour was essential for normalizing the perceived intensity of vibrations across the selected frequency range, while the maximum stimulus duration was required to establish the duration limits in the user interface. An application was developed in MATLAB’s App Designer to control the vibration frequency and signal amplitude, as illustrated in Fig. 3a. The signal amplitude knob was configured with appropriate limits for each frequency to ensure precise adjustments during the evaluation. Both the laptop and amplifier volumes were set to 50%.

The minimum signal amplitude at which vibrations were perceived was determined using a *one down–one up* staircase procedure. Participants were seated in the chair and instructed to gently rest the index finger of their left hand on the 3D-printed pad of the vibrotactile interface, as shown in Fig. 1. The stimulus duration was set to 500 ms, and the initial signal amplitude was adjusted to produce comfortably perceivable vibrations based on participant feedback.

The signal amplitude was then reduced by half of its current value (e.g., an initial amplitude of 1 was followed by 0.5, then 0.25, and so on) until the participant reported no longer perceiving the vibrations. Subsequently, the signal amplitude was increased in the same manner (e.g., from 0.25 to 0.375, then to 0.5625, and so on) until the vibrations were perceived again. This procedure was repeated until five reversals were recorded. Afterward, the amplitude adjustments were made in smaller steps corresponding to the lowest resolution possible on the amplitude knob.

In total, twenty measurements were collected for each frequency from each participant. The median of the final ten measurements was calculated and recorded as the intensity perception threshold for each frequency and participant.

**Fig. 4:**
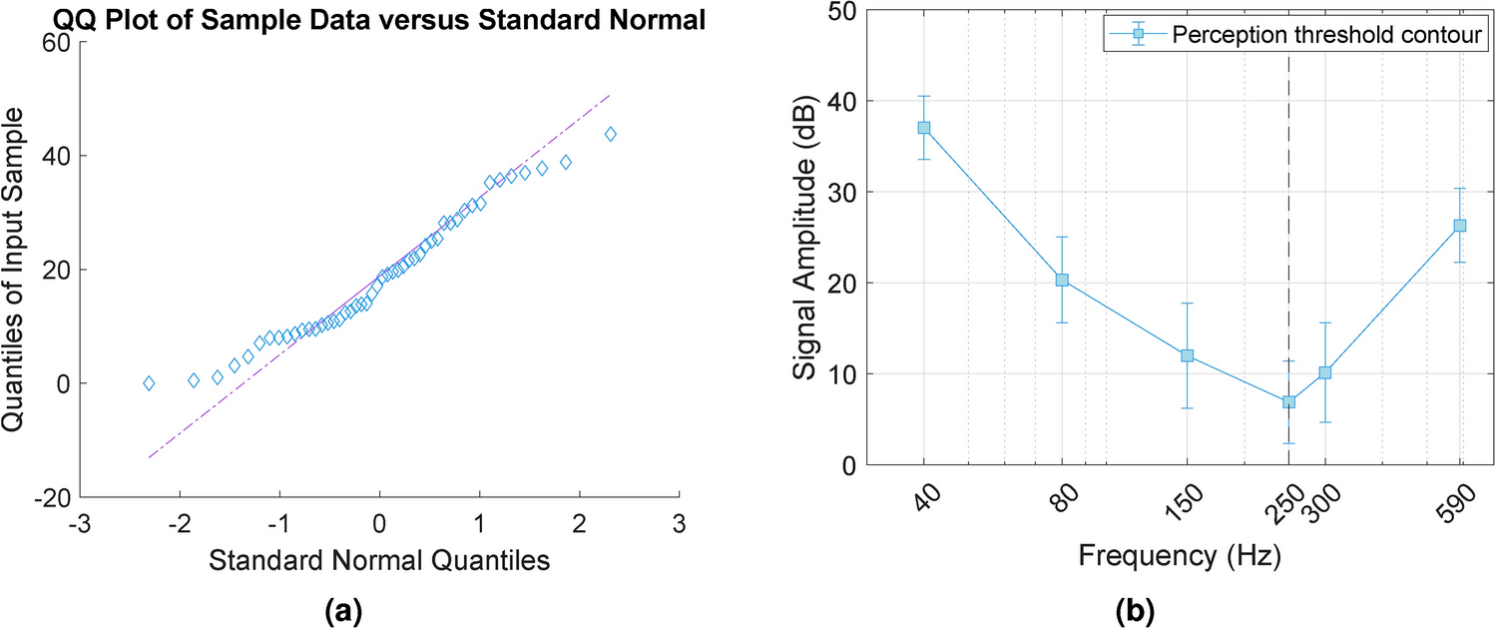
(**a**) Q–Q plot of perception thresholds of 8 participants, reported for each frequency. (**b**) Mean perception thresholds. Error bars represent the standard deviation. The dashed line indicates the peak frequency at 250 Hz corresponding to the lowest recorded signal amplitude taken as reference.

The collected data were analyzed for normality using a Q–Q plot, as shown in Fig. 4a. The perception thresholds reported by the eight participants displayed an approximately normal distribution. This was further confirmed using the Shapiro–Wilk test, which indicated that the data followed a normal distribution.

The threshold contour was computed as the mean of all reported thresholds. Signal amplitude was converted to decibels using Eq. 1^30^, with the reference signal amplitude ($I_{ref}$) defined as the lowest measured value at the peak frequency of 250 Hz.

1 $$\begin{aligned} I_{\text {dB}} = 20 \log \left( \frac{I}{I_{ref}} \right) \end{aligned}$$

Where:

- $I_{dB}$: is the signal amplitude expressed in decibels (dB).
- $I$: is the measured signal amplitude.
- $I_{ref}$: is the reference signal amplitude.

Figure 4b presents the resulting perception threshold contour. As shown, the contour exhibits a U-shape characteristic of the RA-II channel response, aligning with findings reported in the literature^14,31^. The signal amplitudes for each frequency in the main experiment were derived from this intensity perception threshold contour.

After determining the intensity perception threshold contour, we identified an appropriate maximum stimulus duration for the main experiment. Vibrations with durations of 200, 500, and 1000 ms were presented to the participants. Based on their feedback, a duration of 200 ms was sufficient to evoke robust vibration sensations across all tested frequencies. Consequently, this duration was selected as the maximum stimulus duration for the main experiment.

#### Main experimental procedure

Participants were seated in the chair and instructed to gently place the fingertip of their left index finger on the actuator pad, with both arms comfortably resting on the table. Following the procedure proposed by Cohen and Kirman^26^, the vibration amplitude was initially set to 15 dB above the intensity perception threshold, i.e., the threshold contour calculated in Section 3.5 was uniformly shifted upward by 15 dB while maintaining its original shape. This adjustment, grounded in the theory of contours of equal sensation magnitude^13^, aimed to ensure proper discrimination of vibration frequencies^32^. If a participant reported difficulty perceiving the vibrations with this 15 dB increase, the vibration intensity was further adjusted using the amplifier’s volume knob, thereby incrementally shifting the intensity perception threshold contour upward until the vibrations were perceived clearly and comfortably.

**Fig. 5:**
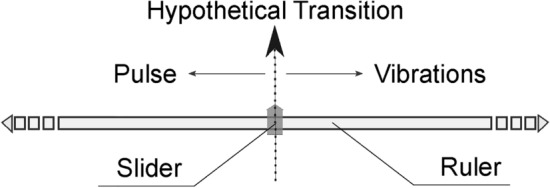
Diagram used to explain the participant what they had to look for in the experiment.

The mouse was controlled with the participant’s dominant hand to ensure precision when calibrating the stimulus duration using the ruler in the user interface, as shown in Figs. 3b and 5. Figure 2b illustrates a participant during the experimentation process. Before starting the main experiment, participants were familiarized with the interface through a training session that included examples of both pulse and vibration sensations.

The user interface was designed for simplicity, featuring a MATLAB slider object representing the stimulus duration and two buttons: one to initialize the experiment and another to save the response and trigger the subsequent stimulus, as shown in Fig. 3b. To avoid response bias, the slider’s ruler was not graduated, and its limits were set between $min=0$ and $max=200$ ms, consistent with the maximum stimulus duration established in the pilot experiment.

Participants were instructed, using a diagram depicted in Fig. 5, to adjust the slider until locating the transition point. This point was defined as the position where moving the slider slightly to the left resulted in the perception of a pulse, and moving it slightly to the right produced the sensation of vibrations. Each frequency was presented five times in random order.

The entire experimental procedure, including the training session, lasted approximately 60 minutes. At the conclusion of the experiment, participants answered an open-ended question about the difficulty of the task.

## Results

**Fig. 6:**
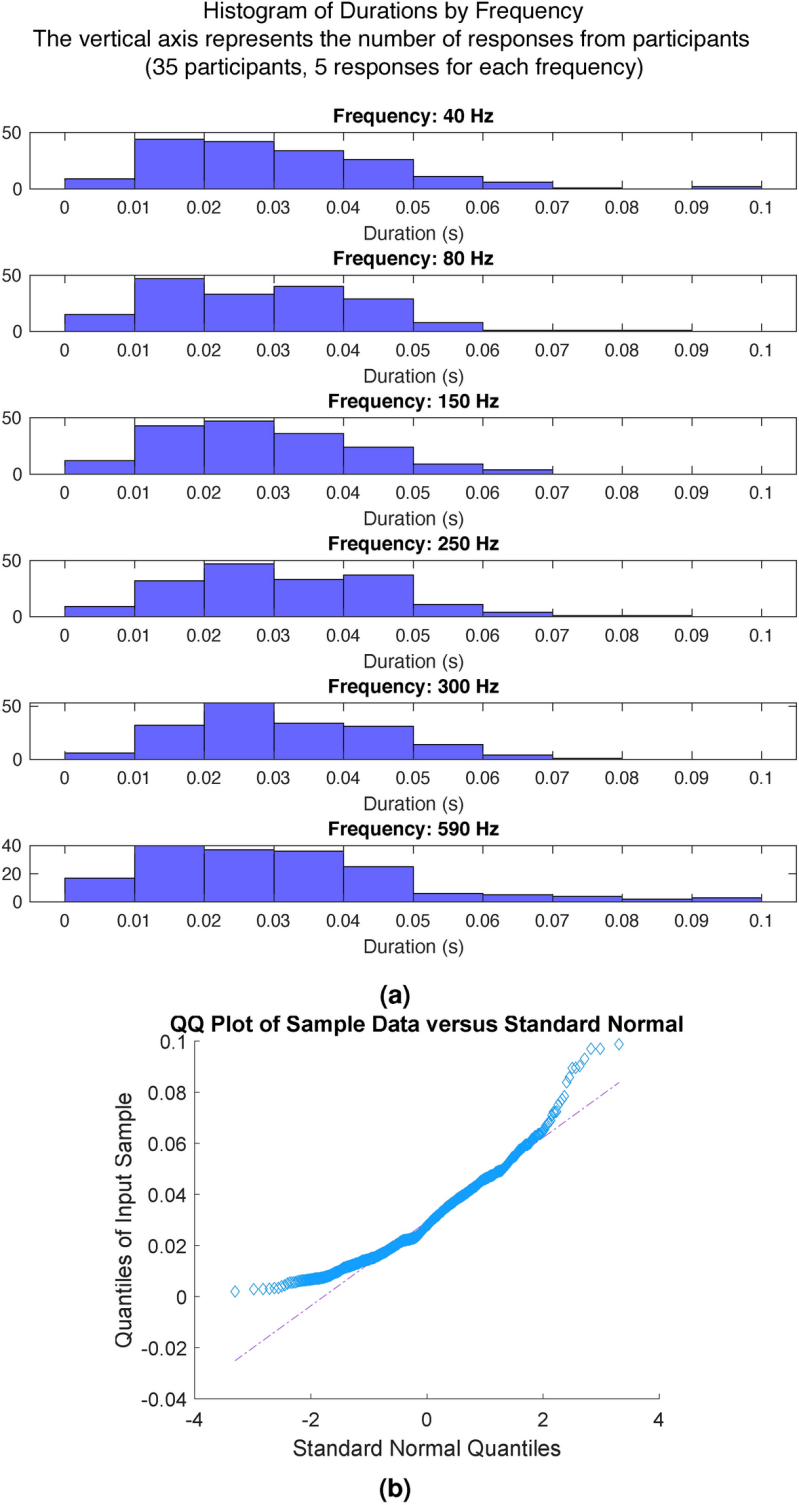
(**a**) Histogram of minimum durations reported by frequency. (**b**) Median minimum duration when participants reported the transition from pulse to vibration sensations. Error bars represent the interquartile ranges.

The minimum durations reported by 35 participants, with five responses recorded for each of the six frequencies, are displayed in the histogram in Fig. 6a. The distribution of the data is further analyzed using a q–q plot shown in Fig. 6b. From the plot, it is evident that the data distribution deviates from a standard normal distribution. The Shapiro–Wilk test confirmed that the data is not normally distributed. Consequently, non-parametric statistical methods were employed for subsequent analyses.

**Fig. 7:**
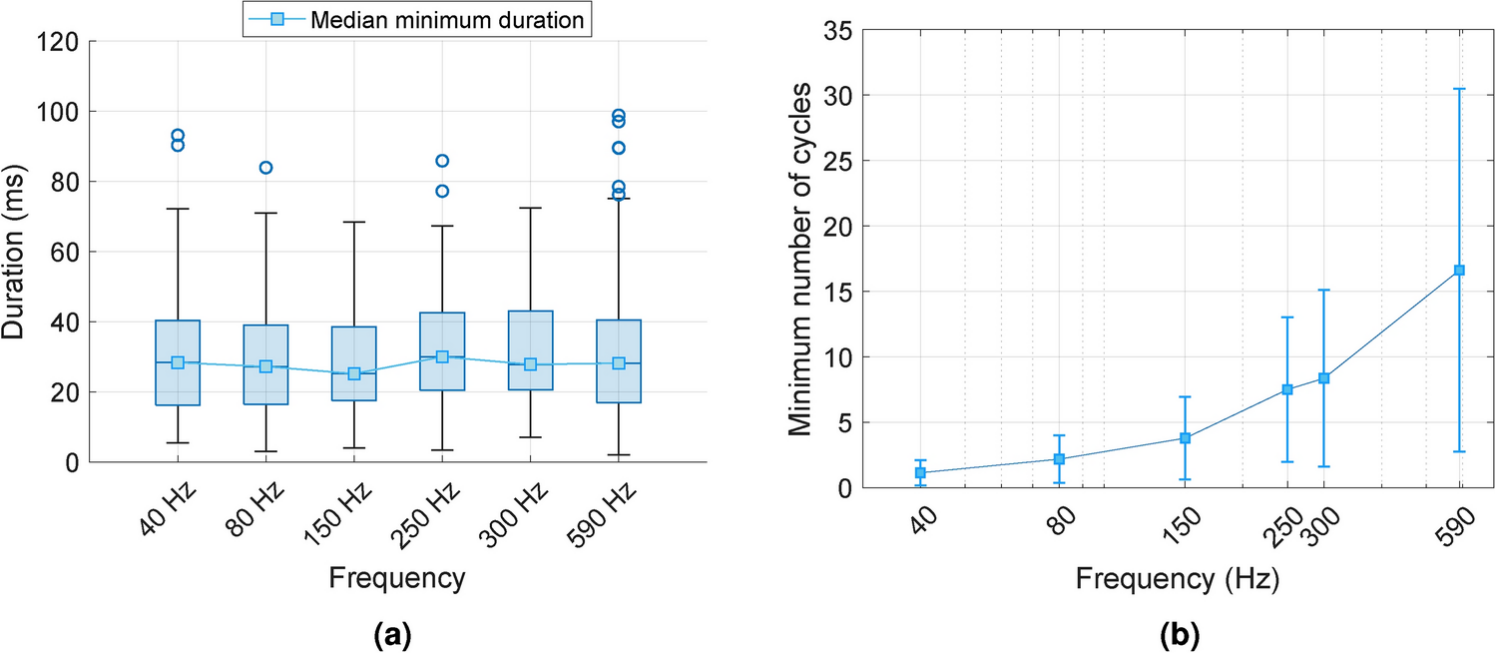
(**a**) Median minimum duration when participants reported the transition from pulse to vibration sensations. Error bars represent the interquartile ranges. (**b**) Median minimum number of cycles (cycle fraction) required to perceive vibration sensations. Error bars represent the interquartile range.

The median minimum durations for each frequency are presented in Fig. 7. A Friedman test for repeated measures was conducted to compare the medians across the selected frequency range. The results revealed a statistically significant difference between medians of minimum duration required to perceive the transition from pulse to vibration sensations ($\chi ^2= 53$, *p* = 3.37 × 10^−10^). However, the effect size was minimal, as indicated by Kendall’s coefficient ($W=0.061$), meaning that the frequency had little effect on this difference.

Additionally, pairwise comparisons were conducted using Wilcoxon Signed-Rank tests, with *p*-values adjusted through the Bonferroni-Holm correction method. The results, summarized in Table 1, demonstrate a statistically significant increase in the minimum duration within the frequency range of 250 to 300 Hz.

**Table 1:** Corrected *p*-values from pairwise comparison between minimum stimuli duration by frequency, using the Wilcoxon Signed-Rank test and the Bonferroni--Holm method.

Frequency (Hz)	80	150	250	300	590
40	0.502	0.838	0.234	0.502	1.458
80	_	1.458	1.211 × 10^−5^*	1.285 × 10^−4^*	0.357
150	_	_	7.276 × 10^−6^*	5.498 × 10^−4^*	0.45
250	_	_	_	1.113	0.234
300	_	_	_	_	0.234

*Indicate statistically significant differences

The results can also be expressed in terms of the minimum number of cycles required for participants to perceive the transition. These values are presented in Table 2 and visualized in Fig. 7. Due to the approximately constant minimum duration, the number of cycles increases as the vibration frequency rises. Notably, at a frequency of 40 Hz, most participants reported the transition point at fewer than two cycles, with a median value of 1.13 *cycles*. In addition, as the frequency increases, the number of cycles grows.

**Table 2:** Median minimum durations, interquartile ranges, and minimum number of cycles by frequency.

	40 Hz	80 Hz	150 Hz	250 Hz	300 Hz	590 Hz
Minimum duration, MD (ms)	28	27	25	30	28	28
IQR (ms)	24	23	21	22	23	24
Minimum number of cycles ($duration*frequency$)	1.13	2.18	3.78	7.5	8.35	16.61

All participants reported that identifying the transition from a ’pulse’ sensation to a ’vibration’ sensation was challenging, particularly when dealing with low-frequency vibrations, i.e., 40–80 Hz.

## Discussion

In this study, the authors present the findings of a controlled experiment investigating the minimum vibrotactile stimulus duration required for perceiving the transition from pulse to vibration sensations. The selected frequency range, from 40 to 590 Hz, primarily activates the RA-II or Pacinian psychophysical channel, which is responsible for vibration sensations.

Initially, the intensity perception thresholds for the tested frequencies were determined, resulting in a U-shaped contour consistent with those reported in the literature^13,14^. This contour was then shifted 15 dB upward and calibrated based on participant feedback to ensure consistent perception of vibrations.

In the main experiment, participants reported minimum durations ranging from 25 to 30 ms across the investigated frequency range. The effect of frequency on the statistically significant differences between median minimum durations at 250 and 300 Hz was very low, suggesting that the minimum duration may not depend strongly on vibration frequency. These findings contrast with results in the auditory modality reported by Doughty and Garner^24^, who observed a minimum stimulus duration of 4.1 ms for a peak frequency of 4000 Hz, with durations decreasing as frequency increased. This discrepancy may arise from the tactile modality’s lower resolution compared to audition^14^ and the multichannel nature of touch^1^. However, the minimum durations found in this study align closely with the 50 ms stimulus duration reported by Kobayashi for perceiving vibrations in consecutive stimuli^27^. Furthermore, Cohen and Kirman^33^ observed that the ability to discriminate between vibration frequencies remains constant within a range of 50–200 ms, which also contrasts with auditory findings.

At the lower end of the frequency range, particularly at 40 Hz, most participants identified the transition point from pulse to vibration sensations at approximately 28 ms, corresponding to 1.13 *cycles*. This suggests that one full cycle plus a fraction is sufficient to elicit vibration sensations. The reduced number of cycles and the complexity of the associated psychophysical processes may explain why participants reported greater difficulty in identifying the minimum duration at lower frequencies. Further research is recommended to confirm and expand upon these findings.

## Conclusions

The findings of this investigation indicate that individuals perceive a transition from pulse to vibration sensations at an approximately constant minimum vibrotactile stimulus duration across the frequency range of 40 to 590 Hz. This result contrasts with findings reported in the auditory modality but aligns closely with prior observations in the tactile domain.

The observation of an approximately constant minimum duration for this transition is particularly advantageous in applications where eliciting vibration sensations while varying frequency is essential, such as in the field of musical haptics^34^. Other potential applications include gaming and virtual/augmented reality, where vibrotactile interfaces may need to evoke either pulse or vibration sensations using tactile illusions, such as phantom motion (vibrations)^35,36^ or the cutaneous rabbit effect (pulses)^37,38^. Minimizing the duration required to elicit vibration sensations contributes to reducing the energy consumption of vibrotactile interfaces, enhancing their efficiency and sustainability.

This study is the first to report on the minimum duration necessary for perceiving the transition from pulse to vibration sensations. Future research could explore additional factors, such as testing other body locations, varying contact areas, investigating lower frequencies to activate the RA-I channel and evoke fluttering sensations, experimenting with different or more complex waveforms, and examining transitions from pulse to vibrotactile pitch and from vibrotactile pitch to pure vibrations.

## Data Availability

Data that supports this investigation is freely available in the following repository: https://github.com/PaulRemache/minimumduration.
